# Bioactivation of Konjac Glucomannan Films by Tannic Acid and Gluconolactone Addition

**DOI:** 10.1021/acsami.4c09909

**Published:** 2024-08-20

**Authors:** Beata Kaczmarek-Szczepańska, Lidia Zasada, Ugo D’Amora, Anna Pałubicka, Anna Michno, Anna Ronowska, Marcin Wekwejt

**Affiliations:** † Department of Biomaterials and Cosmetics Chemistry, Faculty of Chemistry, Nicolaus Copernicus University in Torun, Gagarina 11, 87-100 Torun, Poland; ‡ Institute of Polymers, Composites and Biomaterials, National Research Council, v.le J.F. Kennedy 54, Mostra d’OLtremare Pad. 20, 80125 Naples, Italy; § Department of Laboratory Diagnostics and Microbiology with Blood Bank, Specialist Hospital in Kościerzyna, Alojzego Piechowskiego 36, 83-400 Kościerzyna, Poland; ∥ Department of Laboratory Medicine, Medical University of Gdańsk, Marii Skłodowskiej-Curie 3a, 80-210 Gdańsk, Poland; ⊥ Department of Biomaterials Technology, Faculty of Mechanical Engineering and Ship Technology, Gdańsk University of Technology, Gabriela Narutowicza 11/12, 80-229 Gdańsk, Poland; # Laboratory for Biomaterials and Bioengineering (CRC-Tier I), Dept Min-Met-Materials Eng & Regenerative Medicine, CHU de Quebec, Laval University, Quebec City, Quebec G1 V 0A6, Canada

**Keywords:** wound dressing, bioactivation, konjac glucomannan, tannic acid, gluconolactone, bioactive properties

## Abstract

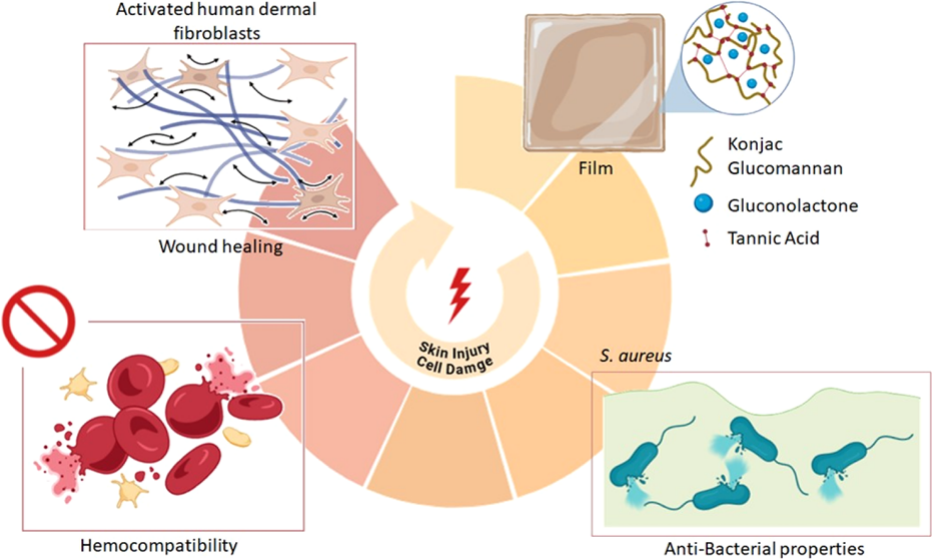

Wound healing is a dynamic process that requires an optimal extracellular environment, as well as an accurate synchronization between various cell types. Over the past few years, great efforts have been devoted to developing novel approaches for treating and managing burn injuries, sepsis, and chronic or accidental skin injuries. Multifunctional smart-polymer-based dressings represent a promising approach to support natural healing and address several problems plaguing partially healed injuries, including severe inflammation, scarring, and wound infection. Naturally derived compounds offer unique advantages such as minimal toxicity, cost-effectiveness, and outstanding biocompatibility along with potential anti-inflammatory and antimicrobial activity. Herein, the main driving idea of the work was the design and development of konjac glucomannan d-glucono-1,5-lactone (KG) films bioactivated by tannic acid and d-glucono-1,5-lactone (GL) addition. Our analysis, using attenuated total reflectance-Fourier transform infrared, atomic force microscopy, and surface energy measurements demonstrated that tannic acid (TA) clearly interacted with the KG matrix, acting as its cross-linker, whereas GL was embedded within the polymer structure. All developed films maintained a moist environment, which represents a pivotal property for wound dressing. Hemocompatibility experiments showed that all tested films exhibited no hemolytic impact on human erythrocytes. Moreover, the presence of TA and GL enhanced the metabolic and energetic activity in human dermal fibroblasts, as indicated by the MTT assay, showing results exceeding 150%. Finally, all films demonstrated high antibacterial properties as they significantly reduced the multiplication rate of both *Staphylococcus aureus* and *Escherichia coli* in bacterial broth and created the inhibition zones for *S. aureus* in agar plates. These remarkable outcomes make the KG/TA/GL film promising candidates for wound healing applications.

## Introduction

As the largest organ in direct contact with the external environment, the skin is highly susceptible to damage from burns, unintentional injuries, and chronic ulcers. The human body, particularly in adulthood, often struggles to heal significant dermal wounds autonomously. As a consequence of an inappropriate or delayed treatment, a great number of negative effects can arise, including severe bleeding, bacterial infections, scarring, tissue necrosis, acute renal failure, and even death.^1^ Therefore, wound healing poses an urgent concern. Maintaining the integrity and regular physiological function of the skin is crucial to preserving patients’ quality of life. At biological and biomolecular levels, wound healing is a complex and well-regulated process in which different phases, such as hemostasis, cell migration, proliferation, and extracellular matrix (ECM) remodeling, play a key role.^2^ Facing each event of this pathological process is of paramount importance to address skin tissue regeneration in a customized way. Indeed, while common medical wound dressings (i.e., gauzes or nonwoven fabrics) can only protect wounds from further mechanical injuries and infections, they often lack antimicrobial, anti-inflammatory, antioxidant, and pro-regenerative properties. Consequently, numerous smart wound dressings, such as modified gauze, foams, injectable formulations, nanofibers, membranes, films, and scaffolds,^3−5^ with controlled properties, have been designed to enhance these essential healing attributes. The most promising biomaterials for wound dressings are hydrogels. This type of material has acquired significant interest in recent years because it resembles natural tissue in terms of ECM structure. Hydrogels can absorb exudates, relieve pain, facilitate gas exchange (CO_2_, O_2_),^6^ and allow the release of growth factors and bioactive agents. Additionally, they may possess self-healing, injectability, and tunable mechanical characteristics, making them highly adaptable to various medical applications. Furthermore, they can be processed by a wide range of technologies, including bioprinting to obtain three-dimensional (3D) dressings able to cover irregular wounds.^7^ Among the hydrogels, polysaccharide-based ones (e.g., hyaluronic acid, gellan gum, chitosan, cellulose, konjac glucomannan (KG) and their derivatives) showed great promise in this field due to excellent biocompatibilities, outstanding bioactive properties, and adequate mechanical strength.^2^ In particular, KG, which is extracted from the konjac plant, belonging to the *Araceae* plant family, is emerging as a sustainable material for wound dressing.^3^ It exhibits high viscosity, solubility in aqueous solutions, swelling ability, and good film-forming properties. Nevertheless, its application is restricted by its weak stability in an aqueous environment due to the high number of hydroxyl (OH) groups present on the polymer chain. Therefore, neat KG does not fully meet all of the criteria required for the design of wound dressings, necessitating further modifications. The first approach involves the use of different polymers (i.e., silk fibroin (SF), xanthan gum (XG), chitosan), while the second one entails the addition of various compounds that act as cross-linkers, forming new bonds between functional groups present in polymer chains. Such modifications improve the stability of the materials and allow their employment as biocompatible coatings or films.^8^ For example, chitosan and KG were processed to create a new bilayered film.^9^ This product demonstrated good biocompatibility and mechanical and barrier properties suitable for wound dressings. KG was also used with γ-poly(glutamic acid) and poly(vinyl alcohol) by following a repeated freeze–thaw method to obtain bilayered hydrogels with tunable porosity in the bottom and upper layer to ensure exudate and antibacterial ability, respectively. Indeed, the macropores in the lower layer were able to absorb a large amount of exudate from the wound; meanwhile, the small pores prevented the bacteria entrance, conferring bacterial-barrier property. Furthermore, the hydrogel displayed excellent swelling ratio and water retention capacity.^8^ XG/KG blend was also used to create a wound dressing without the use of chemical cross-linkers.^10^ XG conferred the gel-like behavior at temperatures around 37 °C, which allowed the production of an in situ forming hydrogel. The hydrogels displayed a transparent and moisturized appearance, allowing continuous monitoring of the wound without dressing removal. By increasing the polymer concentrations from 1 to 2% w/v, the swelling ratio of hydrogel formulations increased, as a result of an increased presence of OH, amino (NH_2_), and carboxyl groups (COOH) at a material’s surface which can easily interact with water molecules. For the same reason, the hydrogels exhibited excellent bioadhesion and cohesiveness. Biological analyses showed that fibroblasts were viable and migrated at 24 and 72 h. Furthermore, results highlighted that the products resulting from XG/KG polysaccharide degradation (e.g., residues of glucose, mannose, glucuronic acid, and glucomannan) encouraged cell migration. Indeed, as demonstrated by Shahbuddin et al., the KG may interact with growth factor receptors available on fibroblast membranes, stimulating their proliferation.^11^ More recently, Xu et al. successfully fabricated an antibacterial drug-loaded composite hydrogel, through a Schiff base reaction between carboxymethyl chitosan and oxidized KG, followed by the encapsulation of stevioside-stabilized honokiol micelles.^12^ The hydrogel exhibited several favorable properties, including a short gel time (<10 min), high water content (>92%), injectability, good adhesiveness, self-healing ability, and high transparency. It also highlighted high biocompatibility, antibacterial and antioxidant properties, and a hemolysis ratio lower than 5%. Furthermore, in vivo results proved that it could speed up wound healing by controlling inflammation and improving re-epithelization.^12^ Few attempts have been devoted to the second approach, which involves adding effective components in pure KG to enhance its ability to control inflammation and bacterial infection while at the same time conferring suitable physicochemical properties (i.e., appropriate residence time and stability). Nowadays, antibiotics are used in about 50% of commercial wound dressings to stop bacterial infections. However, overuse of antibiotics can lead to other issues such as toxicity, allergies, and bacterial resistance.^13^ Furthermore, during the inflammatory phase of wound healing, immune cells can release pro-inflammatory cytokines, which stimulate inflammatory cells to produce reactive oxygen species (ROS) such as superoxide anion radical, hydroxyl radical (^•^OH), and hydrogen peroxide (H_2_O_2_).^14^ Overproduction of ROS in an infected wound can compromise the antioxidant defense mechanism and amplify the inflammatory response, impeding healing and causing scarring that impairs skin function and appearance. Investigating substances with both antibacterial and antioxidant properties is therefore crucial for effective skin healing. Due to their low toxicity, natural plant compounds are considered a reliable source of antibacterial agents.^15^ For example, owing to its in vitro nontoxicity and stability under physiological conditions, materials based on tannic acid (TA) have shown promising potential in tissue regenerative medicine.^16^ Due to its phenolic hydroxyl group and abundance of dihydroxy phenyls, TA can easily bind to other materials through noncovalent interactions, particularly hydrogen bonding and ionic coordination, acting as a cross-linker and conferring antibacterial, anti-inflammatory, and free radical scavenging properties. Furthermore, TA’s phenolic hydroxyl group interacts with blood proteins and peptides to promote blood coagulation.^17^d-Glucono-1,5-lactone, commonly known as gluconolactone (GL), is a cyclic ester (lactone) derivative of d-gluconic acid; as a polyhydroxy acid (PHA) or an oxidized derivative of glucose, GL is found in a wide range of living organisms including humans and microbes. It is an antioxidant molecule capable of protecting against the effects of free radicals brought on by ultraviolet-B (UVB) damage due to its OH groups on several carbons.^18^ Its structure combines the unique features of glucose with the characteristics of α-hydroxy acids, which help prevent water diffusion and evaporation, thereby reducing transepidermal water loss. For this reason, GL has been considered in dermatology and cosmetics for the treatment of dry and sensitive skin.^19^ Additionally, it also has been found to have positive effects against acne.^20^ Further, GL is well known for its capacity to increase the production of collagen and elastin, and improve skin texture uniformity by encouraging cell renewal.^18^ Its protective effects can be correlated to its ability to chelate molecules and its strong antioxidant effects.^21^ Finally, evidence has shown that GL may exert protective effects against other different pathologies, such as myocardial infarction and Ischemia/reperfusion (I/R) injury.^22^

Herein, the main driving idea of the work was the design and development of novel konjac glucomannan films bioactivated by tannic acid and gluconolactone as a smart wound dressing. The films were fully characterized in terms of physicochemical, structural, hemo- and cytocompatibility, and antimicrobial properties. The hypotheses of the study were that (i) the physicochemical properties of KG/TA/GL films can be modulated by varying the ratio between KG and TA, and (ii) the biocompatibility and antibacterial properties can be optimized by adjusting the KG/TA ration as well as the GL concentration.

## Experimental Section

### Chemicals

#### For Film Preparation

Konjac glucomannan (KG) and tannic acid (TA, *M*_w_ = 1701.23 g/mol) and gluconolactone (GL) (99%, *M*_w_ = 178.14 g/mol) were purchased from Pol-Aura (Zawroty, Poland).

#### For In Vitro Studies

Primary human adult dermal fibroblasts (hFB; PCS-201–012), *Staphylococcus aureus* (25923) and *Escherichia coli* (25922) were from the American Type Culture Collection (ATCC, Manassas, VA). MTT (3-(4,5-dimethylthiazol-2-yl)-2,5-diphenyltetrazolium bromide) assays, culture medium (Dulbecco’s modified Eagle’s medium; DMEM), l-glutamine, streptomycin, penicillin, fetal bovine serum (FBS), Trypticase Soy Broth, and Triton X-100 were purchased from Merck (Darmstadt, Germany).

#### Other Chemicals

Diiodomethane (99%) was supplied from Sigma-Aldrich (Poznań, Poland). Glycerine (pure for analysis) was purchased from Avantor Performance Materials Poland S.A. (Gliwice, Poland).

## Materials Preparation

KG and TA were dissolved in 0.1 M acetic acid at 1% concentration, and gluconolactone was separately dissolved in distilled water at 2% w/v concentration. These solutions were mixed in the weight ratio of 80KG/20TA and 50KG/50TA, both with and without the addition of GL (2 and 5% w/w). The mixtures were stirred on a magnetic stirrer for 1 h and placed onto a plastic holder (40 mL per 10 cm × 10 cm). Thin films were obtained by the solvent evaporation process. Pure KG-based films, as well as KG films containing GL, were utilized as controls for comparison (Table 1).

**Table 1:** Nomenclature and Composition of the Different Films

abbreviation	sample
100 KG	film based on konjac glucomannan
100 KG + 2%GL	film based on konjac glucomannan with the 2% w/w addition of gluconolactone
100 KG + 5%GL	film based on konjac glucomannan with the 5% w/w addition of gluconolactone
80KG/20TA	film based on konjac glucomannan and tannic acid in (80/20 w/w%)
80KG/20TA + 2%GL	film based on konjac glucomannan and tannic acid in (80/20 w/w%) with the 2% w/w addition of gluconolactone
80KG/20TA + 5%GL	film based on konjac glucomannan and tannic acid in (80/20 w/w%) with the 5% w/w addition of gluconolactone
50KG/50TA	film based on konjac glucomannan and tannic acid in (50/50 w/w%)
50KG/50TA + 2%GL	film based on konjac glucomannan and tannic acid in (50/50 w/w%) with the 2% w/w addition of gluconolactone
50KG/50TA + 5%GL	film based on konjac glucomannan and tannic acid in (50/50 w/w%) with the 5% w/w addition of gluconolactone

## Materials Characterization

### Attenuated Total Reflectance-Fourier Transform Infrared (ATR-FTIR)

The infrared spectra of the films were obtained using a Nicolet iS5 spectrophotometer (Thermo Fisher Scientific, Waltham, MA) equipped with an ID7 ATR accessory containing a ZnSe crystal at room temperature in ambient air. The following operating parameters were employed: 4 cm^–1^ resolution, 32 scans, and a wavenumber range of 4000–400 cm^–1^.

### Atomic Force Microscopy (AFM)

Surface roughness was assessed using images captured by a microscope equipped with a scanning probe from Veeco Metrology, Inc. (Santa Barbara, CA), operating as a NanoScope IIIa MultiMode Scanning Probe Microscope in tapping mode at room temperature in ambient air. Two parameters, root-mean-square (*R*_q_) roughness and arithmetic mean (*R*_a_), were determined by using Nanoscope Analysis v6.11 software from Bruker Optoc GmbH (Ettlingen, Germany).

### Water Content

The water content of the films was gravimetrically measured by means of an oven-drying method.^23^ Preweighed samples were dried at 105 °C until they reached a constant weight. The results are presented as grams of water per 100 g of a dry sample (*n* = 5).

### Surface Free Energy

The contact angles of glycerin (IFT = 62.7 mN/m) and diiodomethane (IFT = 50 mN/m) on the films were determined using a goniometer fitted with a drop shape analysis system (DSA 10 Control Unit, Krüss, Germany) at ambient temperature in air. Subsequently, the contact angle measurements for both liquids were employed to determine the surface free energy of the material (identified as “s”) (IFT(s)) along with its polar (IFT(s,P)) and dispersive (IFT(s,D)) components, utilizing the Owens-Wendt approach.^24^

### Hemocompatibility

The compatibility of the films was tested with human red blood cells (RBCs) derived from buffy coats, a byproduct of whole blood fractionation processed at the Regional Blood Centre in Gdask (the institutional permission M-073/17/JJ/11). The blood was obtained from healthy volunteers fully compliant with the Declaration of Helsinki, adhering to protocols approved by the Regional Bank review board, and preserved in standard acid citrate dextrose solutions. Blood Bank standards were followed for RBC fractionation.^25^ Tubes containing 3 × 10^9^ cells/mL of RBCs (1.5 mL) and films (0.5 mm × 0.5 mm; 0.015 ± 0.003 mm thickness; *n* = 3) were incubated at 37 °C for 24 h. Cell counts were conducted by using a Superior CE hemocytometer (Marienfeld, Lauda-Königshofen, Germany). Specimens before the experiment were sterilized using UV light (30 W/m^2^; 30 min). Post incubation, samples were centrifuged at 100*g* (3 min; at room temperature) to sediment the erythrocytes. The extent of hemolysis in the supernatants was quantitatively assessed using a 540 nm wavelength with an Ultrospect 3000 pro spectrophotometer (Amersham-Pharmacia-Biotech, Cambridge, U.K.). For controls, RBCs treated with 0.2% Triton X-100 served as a positive control, indicating 100% hemolysis, whereas those incubated without specimens acted as a negative control. The results were expressed as percentage of hemolysis, and according to the literature, samples resulting in below 2% of the hemolysis were nonhemolytic.^26^

### Cytocompatibility

The cytocompatibility of the films was evaluated using hFB with extracts method. The cells were cultured in a 50/50 mix of Ham’s F12 Medium and DMEM, both free of phenol red, enriched with 1 mM l-glutamine, 0.05 mg of streptomycin, and 50 U of penicillin per 1 mL and 10% FBS. 0.3 mg/mL G418 and 10% FBS. After harvesting, they were seeded at a density of 12 × 10^3^ cells per well in a 96-multiwell plate and cultured at 37 °C in a humidified atmosphere of 5% CO_2_ for 24 h. For extract preparation, each film (0.015 ± 0.003 mm thickness; 6 cm^2^/mL of surface/volume ratio; *n* = 4) was immersed in 2 mL of the medium and incubated for 24 h. Before testing, all films were sterilized via UV light (30 W/m^2^; 30 min). Then, the culture medium was discharged and the extracts were added to the plates. The cell viability was evaluated after 48 h of culture and was assessed using an MTT assay (0.60 mmol/L, 4 h of incubation). Post incubation, formazan crystals were dissolved in a solution of 10% SDS and 50% DMF. The development of the colored product metabolized by living cells was colorimetrically assessed using a microplate reader (Victor, PerkinElmer) at 595 nm toward reference 690 nm. The viability of treated cells was quantified relative to that of untreated control cells at tissue culture plastic (TCP) as 100%.

### Antibacterial Properties

Two antibacterial evaluation methods were utilized: (1) the Kirby–Bauer disk diffusion method^27^ and (2) the McFarland bacterial growth method.^28^ The antibacterial properties of the films were evaluated against *S. aureus* and *E. coli*. For the disk diffusion test, 100 μL of a bacterial suspension with a concentration of 1.5 × 10^8^ CFU/mL was applied to Mueller–Hinton plates. Tested films (15 mm in diameter, 0.015 ± 0.003 mm thickness; *n* = 3) were subsequently placed on these agars. Zones of inhibition were measured (±0.1 mm) after 1, 3, and 7 days of incubation (at 35 ± 1 °C). In the McFarland test, the turbidity of bacterial suspensions starting with a bacterial concentration of 0.5 McFarland index (iMS) was monitored hourly using the DensiChEK Plus device. The films (0.5 mm × 0.5 mm; 0.015 ± 0.003 mm thickness; *n* = 3) were incubated with bacteria suspended in 2 mL of Trypticase Soy Broth. The device’s maximum measuring range was 4 iMS. Before testing, the specimens were sterilized via UV light exposure (30 W/m^2^; 30 min). A control group with bacteria incubated without specimens served as a negative control.

### Statistical Analysis

The data was statistically analyzed using SigmaPlot 14 software (Systat Software, San Jose, CA). All of the results were expressed as mean ± standard deviations (SD) and evaluated by one-way analysis of variance (ANOVA). For each experiment, the number of samples (n) are specified. To check for normal distribution, the Shapiro-Wilk test was employed. For multiple comparisons with the control group, the Bonferroni *t* test was applied, with the statistical significance defined as *p* value (*p*) < 0.05.

## Results and Discussion

### FTIR-ATR

**Figure 1:**
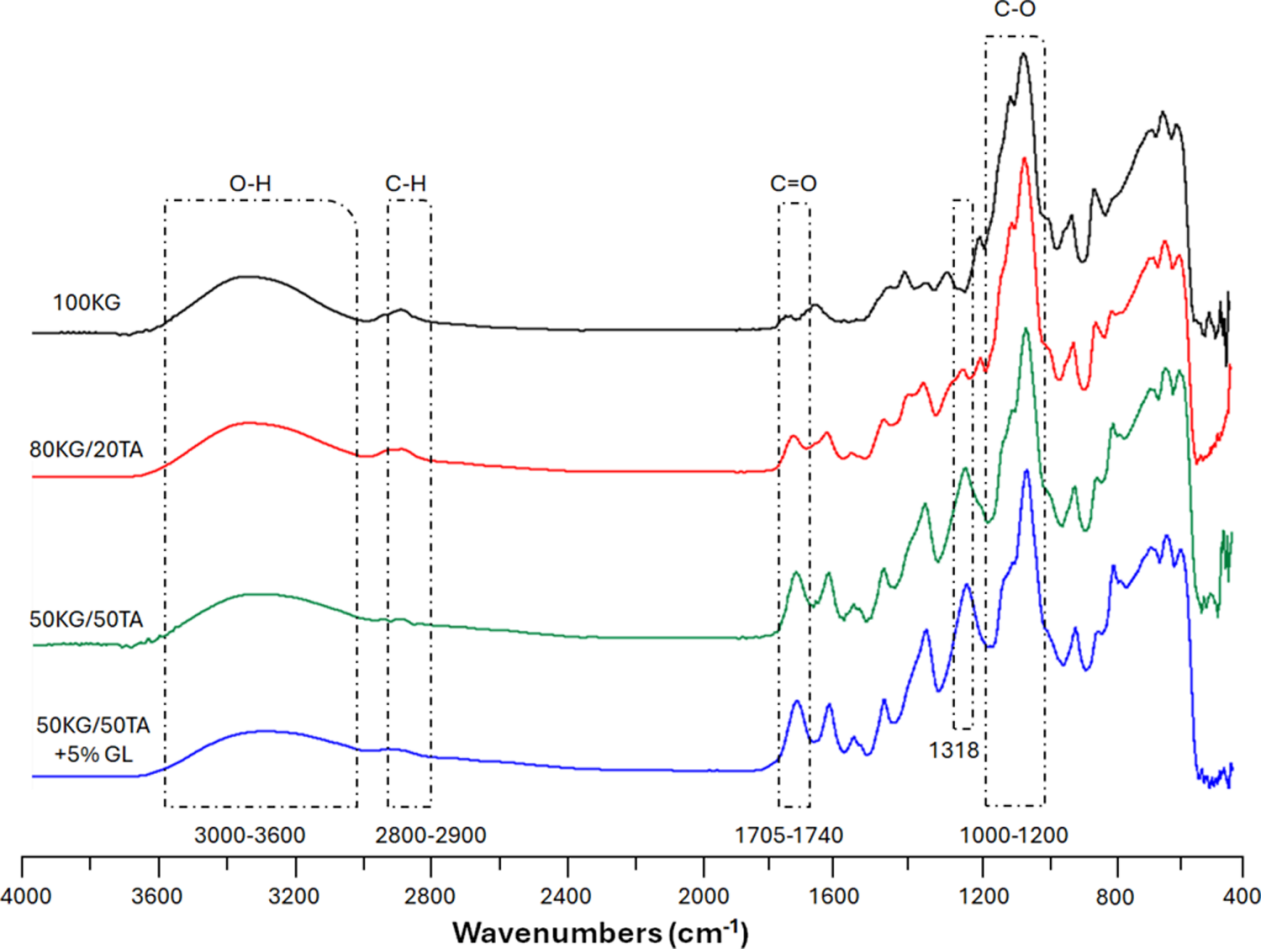
Representative FTIR-ATR spectra of the obtained films between 4000 and 400 cm^–1^.

FTIR spectra of KG films bioactivated by TA and GL were analyzed to determine the structural modifications and interactions (Figure 1). The spectra exhibited broad O–H stretching peaks around 3000–3600 cm^–1^, C–H stretching peaks around 2800–2900 cm^–1^, while the C=O stretching region (1705–1740 cm^–1^) showed new peaks suggesting interactions with tannic acid. The C–O stretching vibrations in the glycosidic linkage region are in the range between 1000 and 1200 cm^–1^. The same range was observed for KG/TA + GL films due to the presence of both phenolic hydroxyl groups and possibly carboxylate groups of GL. Meanwhile, additional peaks corresponding to the phenolic hydroxyls of tannic acid were identified, confirming the successful bioactivation of KG films. Indeed, a peak at around 1318 cm^–1^, corresponding to the C–O stretching vibrations of phenolic hydroxyl groups from tannic acid, was observed.^29^

### Atomic Force Microscopy

AFM was used to assess the films’ morphology. The analysis highlighted the uniform smooth surfaces of all of the investigated films and confirmed the successful combination of the different components. As reported in Table 2, by increasing the TA weight ratio, the films exhibited higher values of *R*_a_ and *R*_q_, spanning from 1.09 nm (*R*_a_) and 1.37 nm (*R*_q_) for 100 KG to 1.49 nm (*R*_a_) and 1.90 nm (*R*_q_) for 80KG/20TA and 1.87 nm (*R*_a_) and 2.29 nm (*R*_q_) for 50KG/50/TA. Meanwhile, the inclusion of GL seemed to slightly decrease the roughness for all of the different compositions (100 KG, 80KG/20TA, 50KG/50TA). Particularly, as also observed by the previous studies, the increased surface roughness may be attributed to the greater amount of TA that interacts with KG through hydrogen-bond interactions.^30^ As a result, there are more exposed functional groups on the film surface, and the polymeric chain orientation is more disordered. For the film 100 KG + 2% GL, the roughness increases compared to that of the 100 KG film, which does not align with the expected trend. Glycerol is known to act as a plasticizer. However, at certain concentrations, glycerol might interact differently, potentially causing phase separation or disrupting the uniformity of the film. This disruption could lead to an increased surface roughness.

**Table 2:** Roughness Parameters and Water Content Values for All Studied Films^a^

specimen	*R*_a_ [nm]	*R*_q_ [nm]	water content [g/100 g]
100 KG	1.09 ± 0.15^bc^	1.37 ± 0.11^bc^	9.17 ± 0.79^bc^
100 KG + 2%GL	1.35 ± 0.07^ac^	1.72 ± 0.06^ac^	10.32 ± 1.14^c^
100 KG + 5%GL	0.78 ± 0.04^abc^	1.01 ± 0.08^abc^	10.11 ± 1.27^c^
80KG/20TA	1.49 ± 0.08^ac^	1.90 ± 0.10^ac^	11.84 ± 1.86^ac^
80KG/20TA + 2%GL	1.31 ± 0.11^ac^	1.56 ± 0.09^bc^	9.56 ± 0.39^bc^
80KG/20TA + 5%GL	1.15 ± 0.07^bc^	1.44 ± 0.06^bc^	10.18 ± 0.78^c^
50KG/50TA	1.87 ± 0.09^ab^	2.29 ± 0.14^ab^	15.79 ± 0.36^ab^
50KG/50TA + 2%GL	1.09 ± 0.10^bc^	1.26 ± 0.09^bc^	11.72 ± 0.31^ac^
50KG/50TA + 5%GL	1.01 ± 0.07^bc^	1.15 ± 0.07^abc^	10.83 ± 0.29^c^

a (*n* = 5; ^a^ significantly different from 100 KG – *p* < 0.05; ^b^ significantly different from 80KG/20TA – *p* < 0.05; ^c^ significantly different from 50KG/50TA).

### Water Content

In wound healing, the ability of the film to interact with water plays a pivotal role, as the wound dressing should be able to ensure a moist environment. Indeed, different studies have proved that moist wounds heal 50% quicker than dry ones.^2^ Films obtained with only KG polymer showed water content values of between 9.17 and 100 g of dry film (Table 2). By increasing the TA amount, the values increased, ranging from 9.17/100g (100 KG) to 15.79/100g (50KG/50TA). This may be ascribed to a greater affinity of the film to water due to a higher presence of OH groups exposed to the polymer matrix. By contrast, to better highlight the effect of GL on water content, two different aspects should be taken into consideration. By comparing 100 KG + 2%GL, 80KG/20TA + 2%GL, and 50KG/50TA + 2%GL, the presence of GL did not significantly influence water content. The same trend was observed for the groups functionalized with 5% GL. Similarly, by comparing 100 KG, 100 KG + 2%GL, and 100 KG + 5%GL as well as 80KG/20TA, 80KG/20TA + 2%GL, and 80KG/20TA + 5%GL, water content did not significantly change. However, at higher TA concentrations, 50KG/50TA showed a significant decrease of water content from 15.79/100g (50KG/50TA) to 10.83/100g (50KG/50TA + 5%GL). This may be correlated to a more compact and denser polymer network, even though GL is widely recognized as a powerful moisturizer in cosmetics. Indeed, according to Pereira et al.,^31^ the water content is related to the total void volume occupied by water molecules in the network microstructure of the film.

### Surface Free Energy

The intrinsic variation in surface energy is among the most important properties that have received poor attention. Compared to the atoms in the bulk, the surface atoms have fewer near neighbors due to their undercoordination. An excess of “unsatisfied bond energy” results from “dangling bonds” that are exposed at the surface of a material. Water interactions, protein adsorption, and cell attachment are controlled by the surface energy or intermolecular connections of biomaterials when they are implanted or encounter biological environments. The control of later proliferation, differentiation, and eventually tissue development at the interface depends on the early adherence of cells to biomaterials. Therefore, the rational design of biomaterials with specified functionalities critically depends on the ability to understand how surface energy influences a surface’s interactions with the biological environment^32^

Herein, the results from surface free energy calculations are summarized in Table 3. The dispersive component was in the range of 23.11–28.99 mJ/m^2^ for all of the samples, and the polar component was in the range of 10.56–18.91 mJ/m^2^. Particularly, for all of the compositions, the inclusion of GL increases IFT(s) and its IFT(s,P) in a concentration-dependent manner; meanwhile, it decreases IFT(s,D). This can be ascribed to the hydrophilic nature of GL.^33^ Similarly, by increasing TA, IFT(s) and IFT(s,P) significantly decreased as a result of the cross-linking process between TA and KG. Consequently, fewer hydrophilic groups were free on the surface.

**Table 3:** Surface Free Energy (IFT(s)), Its Polar (IFT(s,P)), and Dispersive (IFT(s,D)) Components of Films Based on Konjac Glucomannan and Tannic Acid^a^

specimen	IFT(s) [mJ/m^2^]	IFT(s,P) [mJ/m^2^]	IFT(s,D) [mJ/m^2^]
100 KG	41.26 ± 1.48^c^	18.01 ± 0.85^bc^	28.99 ± 0.20
100 KG + 2%GL	43.47 ± 0.49^bc^	18.58 ± 0.30^bc^	25.25 ± 0.63^abc^
100 KG + 5%GL	44.30 ± 1.17^abc^	18.91 ± 0.72^bc^	25.39 ± 0.45^abc^
80KG/20TA	36.84 ± 0.52^a^	10.56 ± 0.24^a^	28.12 ± 0.29
80KG/20TA + 2%GL	37.93 ± 1.03^a^	12.00 ± 0.37^abc^	26.28 ± 0.28^abc^
80KG/20TA + 5%GL	43.87 ± 1.07^abc^	15.75 ± 0.77^abc^	25.94 ± 0.66^abc^
50KG/50TA	36.37 ± 1.76^a^	10.61 ± 0.13^a^	27.96 ± 0.22
50KG/50TA + 2%GL	38.57 ± 0.35^a^	12.10 ± 0.49^abc^	26.92 ± 0.49^ab^
50KG/50TA + 5%GL	39.02 ± 0.86^ac^	13.26 ± 0.68^abc^	23.11 ± 1.08^abc^

a (*n* = 5; ^a^ significantly different from 100 KG – *p* < 0.05; ^b^ significantly different from 80KG/20TA – *p* < 0.05; ^c^ significantly different from 50KG/50TA).

### Hemocompatibility and Cytocompatibility

**Figure 2:**
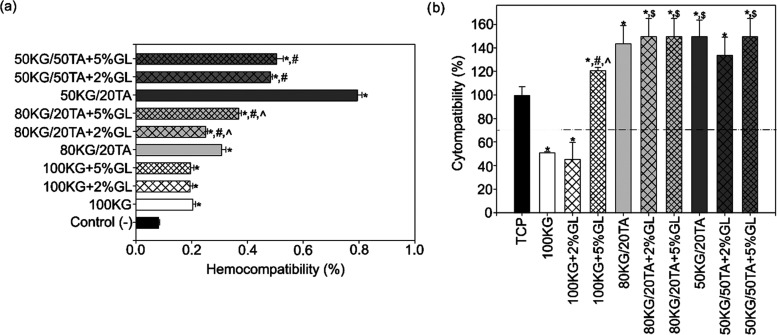
Effect of the developed films on (a) the hemocompatibility of human erythrocytes (hemolysis rate) after 24 h exposure to films and (b) the cytocompatibility of hFB cells after 48 h of culture (*n* = 4; data are expressed as the mean ± SD; * significantly different from the respective controls TCP or (−) (*p* < 0.05), ^#^ significantly different from the respective film group without GL (*p* < 0.05), ^∧^ significantly different from the applied concentration of GL (*p* < 0.05); ^$^ indicates cell viability over 150% with SD ± 10%).

The hemocompatibility experiments (Figure 2b) revealed that all tested films exhibited no hemolytic impact on human erythrocytes (hemolysis degree remaining below 0.8% compared to the positive control); thus, these materials can be classified as nonhemolytic.^26^ Interestingly, films containing TA (80KG/20TA and 50KG/50TA) showed an increase in hemolysis (ca. 0.05–0.6%), while the addition of GL generally reduced this effect, particularly in the 50KG/50TA specimens.

The developed films demonstrated varying levels of cytocompatibility based on their composition (Figure 2a). Most of the specimens can be qualified as noncytotoxic (cell viability exceeded the necessary 70%; compared to TCP^34^). The exception was the 100 KG films, which lacked adequate cross-linking. Additionally, films containing both TA and GL demonstrated increased metabolic and energetic activity in fibroblasts, as indicated by the MTT assay, showing results exceeding 150% (compared to TCP).

KG is widely acknowledged for its biocompatibility and has been extensively utilized in a diverse array of biomedical applications, like drug delivery systems, wound dressings, biological scaffolds, or as a functional ingredient in health supplements.^35^ Moreover, materials based on KG exhibit high antioxidant activity, which allows for the neutralization of free radicals in biological cells and their protection against damage due to oxidative stress.^36^ Further, there are also reports of their anticancer potential. For instance, KG has been shown to have a reversal effect on multidrug resistance in HepG2/5-FU cancer cells by suppressing AKT signaling and increasing p53 expression.^37^ Our films were based on formulation KG/TA, and we used TA, a polyphenol approved by the Food and Drug Administration,^38^ which also has recently gained popularity in biomedical applications.^39^ For their modification, we applied gluconolactone, a polyhydroxy acid, which as well was confirmed to be cytocompatible with various cell lines.^18^

All components were previously studied in various systems, and there are reports indicating that they can be considered biocompatible. For example, the KG-gelatin-matrine hydrogel has demonstrated hemocompatibility (sheep blood, concentration-dependent effect)^40^ as well as cytocompatibility was confirmed for KG-silk fibroin sponges (on human dermal fibroblasts),^41^ KG-chitosan films (on Chinese hamster ovary cells),^9^ or KG-guanosine hydrogel (on L929 fibroblasts).^43^ For TA, biocompatibility has also been demonstrated in numerous developed biomaterials. Examples include sodium alginate-glycerol-TA film (on human embryonic kidney cells),^44^ chitosan-silk fibroin-TA hydrogel (on NIH-3T3 fibroblasts),^45^ and in a biocomposite based on poly(vinyl alcohol)-calcium metaphosphate-TA.^46^ In the case of GL, the literature reports biocompatible materials incorporating it as an additive, such as bone cements,^47^ nanocarriers,^48^ or hydrogels^49^ and also GL is attributed with antioxidant activity.^50^ Furthermore, both components, KG and TA, exhibit significant bioactivity, with noted effects on cellular metabolic activity and proliferation. The effect of stimulating metabolic activity in fibroblasts was confirmed in studies by Huang et al. and Shahbuddin et al., and it was associated with specific sugar receptors on their cell surfaces.^51,52^ While for TA, significant increases in cell proliferation were also found in experiments of Feng et al. and Bai et al.^53,54^ However, some in vitro studies reported a slight reduction in cell viability, for example, for undiluted KG extracts tested on NIH/3T3 fibroblasts.^55^

Our results are mostly consistent with these studies but are also undoubtedly connected with concentration-dependent effects and polymer cross-linking. A notable reduction in cell viability was found for uncross-linked or weakly cross-linked materials (100 KG and 100 KG + 2%GL; Figure 2), and significant improvement in cell metabolic activity was observed in 80KG/20TA and 50KG/50TA films, with and without GL (MTT assay results exceeding 150% compared to the control; Figure 2). These differences arise from the composition and biostability of the films, but other contributing factors could include the source of KG, the extraction process, the purification method, and its molecular weight.^56^ In conclusion, films based on KG/TA and GL exhibit biocompatibility and can be considered to be suitable for medical applications.

### Antibacterial Properties

**Figure 3:**
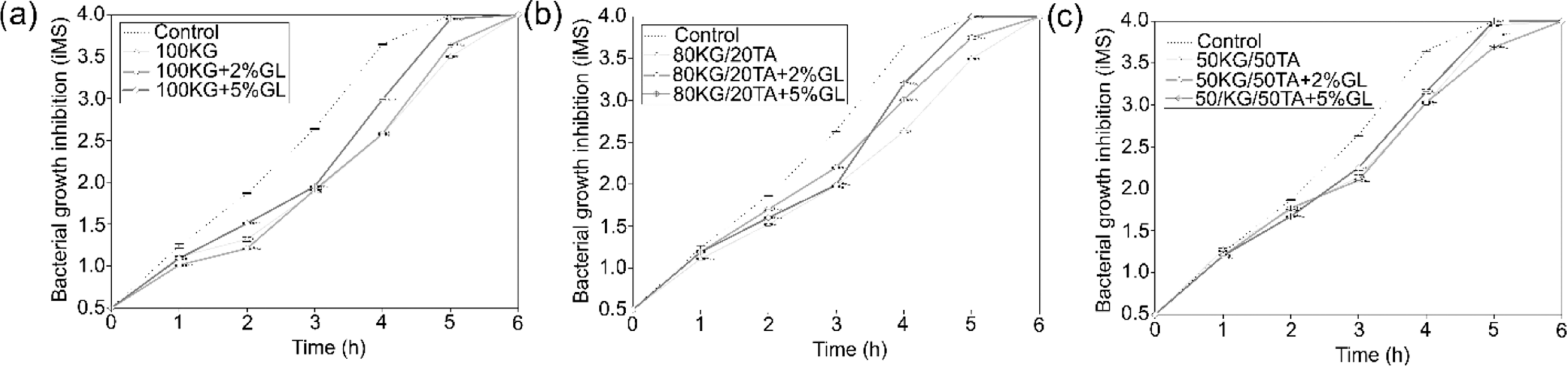
Antibacterial properties of films determined by McFarland index measuring the turbidity of *S. aureus* bacteria broth: (a) 100 KG group, (b) 80KG/20TA group, and (c) 50KG/50TA (*n* = 3; data are expressed as the mean ± SD; statistical analysis available on Table S1).

**Figure 4:**
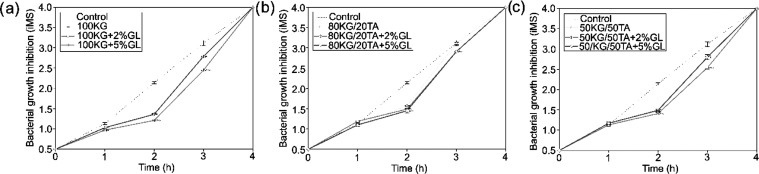
Antibacterial properties of films determined by McFarland index measuring the turbidity of *E. coli* bacteria broth: (a) 100 KG group, (b) 80KG/20TA group, and (c) 50KG/50TA (*n* = 3; data are expressed as the mean ± SD; statistical analysis available on Table S1).

All tested films showed antibacterial properties as they significantly reduced the multiplication rate of both *S. aureus* and *E. coli* in bacteria (Figures 3, 4 and Table S1) and the zone of inhibition in agar plates (Figures S1 and S2).

Analyzing the results of McFarland studies (Table S1), in the case of *S. aureus*, the films 100 KG and 100 KG + 2%GL exhibited the most effective antibacterial properties in the short term (up to 2 h), whereas in the longer term (up to 5 h), the films 80KG/20TA and 50KG/50TA + 2%GL also demonstrated effectiveness. The addition of GL had an adverse effect on the antibacterial properties of the films (Figure 3). However, in the case of *E. coli* (Table S1), films 100 KG and 100 KG + 2%GL showed the most favorable effects in the short term (up to 2 h), while in the longer term (over 3 h), 100 KG + 2%GL and 50KG/50TA + 2%GL were most effective. The addition of GL in the case of *E. coli* did not show a clear trend in its impact on the antibacterial properties (Figure 4).

The results of the bacterial growth inhibition zones were different for both bacteria, and the films only exhibited this zone for *S. aureus* (Table 4, Figures S1 and S2). Those zones were observed 24 h after the films were applied to the agar plates and remained consistent for up to 7 days of observation. It should also be noted that visible discolorations of the substrate (Figures S1 and S2) in the photos do not constitute inhibition zones. Further, it was found that increasing the TA content in the film formulations enhanced the antibacterial properties, and the largest zone was confirmed for the 50KG/50TA films. However, no clear trend was observed regarding the impact of GL on the size of the inhibition zones.

**Table 4:** Kirby–Bauer Zone of Inhibition Test for Films after 1, 3, and 7 Days^a^

	zone of inhibition (mm)					
	*S. aureus* (ATCC 25923)			*E. coli* (ATCC 25922)		
specimen	24 h	72 h	7 days	24 h	72 h	7 days
100 KG		15.0 ± 0.5			15.0 ± 0.5	
100 KG + 2%GL		15.0 ± 0.5			15.0 ± 0.5	
100 KG + 5%GL		15.0 ± 0.5			15.0 ± 0.5	
80KG/20TA		20.0 ± 0.8*			15.0 ± 0.5	
80KG/20TA + 2%GL		22.3 ± 0.9*			15.0 ± 0.5	
80KG/20TA + 5%GL		21.3 ± 1.2*			15.0 ± 0.5	
50KG/50TA		26.3 ± 0.5*			15.0 ± 0.5	
50KG/50TA + 2%GL		26.3 ± 0.5*			15.0 ± 0.5	
50KG/50TA + 5%GL		26.3 ± 0.5*			15.0 ± 0.5	

a (*n* = 3; * significantly different from the KG (*p* < 0.05), ^#^ significantly different from the respective film group without GL (*p* < 0.05), ^∧^ significantly different from the applied concentration of GL (*p* < 0.05)).

Antibiotic resistance is a significant clinical issue despite the widespread use of antibiotics to treat infected wounds. As an alternative, naturally derived bioactive molecules have garnered considerable attention.^43^ It was previously reported that KG and GL exhibit antibacterial properties; however, this activity is classified as relatively low.^58^ Studies by both Chen et al. and Ni et al. demonstrated the absence of antibacterial properties in materials based on KG.^59,60^ But Neto et al. found that the activity of KG is selective and dose-dependent and confirms bacterial inhibition zone against *S. aureus* and *Candida albicans* (but not against *Pseudomonas aeruginosa* and *E. coli*).^9^ For GL, the antibacterial effect is mainly associated with pH reduction and has been confirmed, for example, against *Listeria monocytogenes*.^62^ In contrast, TA exhibits high bactericidal properties and effectively reduces biofilm formation of various strains, including *S. aureus*, *Klebsiella pneumoniae*, *E. coli*, and *Helicobacter pylori*.^63^

Our findings align with the aforementioned reports but also depend on the applied method, formulation, and biostability of the films. The first method analyzes bacterial multiplication over a period of up to 5 h, while the second assesses the inhibition zone over a period of up to 7 days. KG exhibited some antibacterial properties against both tested bacteria in the bacterial broth but did not show an inhibition zone. The combination of both components KG/TA significantly improved antibacterial efficacy in McFarland experiments and for *S. aureus* in Kirby-Bauer, indicating that the addition of TA can be attributed to this effect. No clear trend was observed for GL with regard to these properties. The TA antibacterial property is attributed to the presence of phenolic hydroxyl groups, which may cause the disintegration of the bacterial cell membrane, enzyme complexation, or deprivation of substrates, ions, and minerals.^64^ Further, the lack of long-term antibacterial effectiveness against *E. coli* in all films may be related to the inherent properties of the bacterium itself. *E. coli* is classified as a Gram-negative bacterium, and previous literature has observed a reduced effectiveness of various antibacterial agents against it. This is possibly due to the lipopolysaccharide-rich outer membrane, which prevents the diffusion of these agents into the bacterial cell membrane.^65^ In conclusion, films based on 50KG/50TA + 2%GL exhibit the most promising antibacterial abilities.

## Conclusions

Wound healing is a complex process that often requires a multifunctional smart wound dressing. Therefore, this study successfully designed and developed novel bioactivated konjac glucomannan (KG) films incorporating tannic acid (TA) and gluconolactone (GL), aimed at promoting healing after extensive skin tissue defects. The proposed combination of components offers improved physicochemical and biological properties with high bioactive potential, addressing the critical needs for moisture retention, infection control, and biocompatibility. The presence of TA notably enhanced antibacterial effectiveness, particularly against *S. aureus*, as confirmed by inhibition zone measurements and McFarland bacterial growth studies. All films demonstrated excellent hemocompatibility, exhibiting no hemolytic impact on human erythrocytes. The addition of GL further enhanced the films’ ability to increase metabolic and energetic activity in human dermal fibroblasts, promoting cell proliferation and overall wound healing efficiency. Finally, TA was confirmed as an effective cross-linker for the KG matrix, while GL was loosely embedded within this polymer network. In summary, the developed KG/TA/GL films demonstrate promising potential for dermal healing applications. Their enhanced properties and biocompatibility make them suitable for use as advanced multifunctional dressings, contributing to more effective and sustainable chronic wound care solutions. Future research will focus on clinical evaluations and exploration of the potential of these films in various types of wounds, including burn injuries, sepsis, and chronic wounds, to fully realize their benefits in medical applications.

## Supporting Information Available

Antibacterial properties of films determined by McFarland index measuring the turbidity of *S. aureus* and *E. coli* broth (Table S1); antibacterial properties of films determined by the Kirby–Bauer zone of inhibition of *S. aureus* up to 7 days of incubation (Figure S1); and antibacterial properties of films determined by the Kirby–Bauer zone of inhibition of *E. coli* up to 7 days of incubation (Figure S2) (PDF)

## Abbreviations

KG: konjac glucomannan
TA: tannic acid
GL: gluconolactone
Ra: average roughness
Rq: root-mean-square roughness
IFT(s): the surface free energy
IFT(s,P): polar component
IFT(s,D): dispersive component
