# Supplementary material for: Bioactivation of Konjac Glucomannan Films by Tannic Acid and Gluconolactone Addition
Source: ACS Appl Mater Interfaces. 2024 Aug 20;16(35):46102–12. doi: 10.1021/acsami.4c09909 (PMC11378156; doi:10.1021/acsami.4c09909)
Supplement: Supplementary file 1 — am4c09909_si_001.pdf [file am4c09909_si_001.pdf]

Supporting Information

BIOACTIVATION OF KONJAC GLUCOMANNAN FILMS BY TANNIC ACID AND GLUCONOLACTONE ADDITION

Beata Kaczmarek-Szczepańska*[a], Lidia Zasada[a], Ugo D'Amora[b], Anna Pałubicka[c], Anna Michno[d], Anna Ronowska[d] and Marcin Wekwejt[e,f]

[a] *Department of Biomaterials and Cosmetics Chemistry, Faculty of Chemistry, Nicolaus Copernicus University in Torun, Gagarina 11, 87-100 Torun, Poland*
[b] *Institute of Polymers, Composites and Biomaterials, National Research Council, v.le J.F. Kennedy 54, Mostra d'OLtremare Pad. 20, 80125 Naples, Italy*
[c] *Department of Laboratory Diagnostics and Microbiology with Blood Bank, Specialist Hospital in Kościerzyna, Alojzego Piechowskiego 36, 83-400 Kościerzyna, Poland*
[d] *Department of Laboratory Medicine, Medical University of Gdańsk, Marii Skłodowskiej-Curie 3a, 80-210 Gdańsk, Poland*
[e] *Department of Biomaterials Technology, Faculty of Mechanical Engineering and Ship Technology, Gdańsk University of Technology, Gabriela Narutowicza 11/12, 80-229 Gdańsk, Poland*
[f] *Laboratory for Biomaterials and Bioengineering (CRC-Tier I), Dept Min-Met-Materials Eng & Regenerative Medicine, CHU de Quebec, Laval University, Quebec City, QC G1V 0A6, Canada*

* **Corresponding Author**

beata.kaczmarek@umk.pl

Table S1. Antibacterial properties of films determined by McFarland index measuring the turbidity of *Staphylococcus aureus* and *Escherichia coli* broth (n = 3; data are expressed as the mean ± SD; max. SD ± 0.05 * significantly different from control ($p < 0.05$), # significantly different from the respective film group without GL ($p < 0.05$), ^ significantly different from the applied concentration of GL ($p < 0.05$)).

Specimen	McFarland index (iMS)											
	S. aureus (ATCC 25923)							*E. Coli* (ATCC 25922)				
	0h	1h	2h	3h	4h	5h	6h	0h	1h	2h	3h	4h
Control		1.25	1.86	2.65	3.65	4.0			1.16	2.14	3.13	
100KG		1.11 *	1.32 *	1.89 *	2.57 *	3.50 *			0.96 *	1.28 *	2.78 *	
100KG+2%GL		1.01 *	1.20 *,^	1.91 *	2.58 *,^	3.63 *,#,^			0.97 *	1.21 *,^	2.45 *,#,^	
100KG+5%GL		1.09 *	1.51 *,#,^	1.96 *	3.01 *,#,^	3.94 #,^			1.04	1.36 *,^	2.80 *,^	
80KG/20TA		1.11 *	1.52 *	1.99 *	2.62 *	3.49 *,^			1.11	1.58 *	3.11 *	
80KG/20TA+2%GL	0.5	1.19	1.70 *,#,^	2.13 *,#,^	3.01 *,#,^	3.74 *,#,^	4.0	0.5	1.19	1.49 *	2.91 *,#	4.0
80KG/20TA+5%GL		1.19	1.60 *,^	1.99 *,^	3.20 *,#,^	4.00 #			1.11	1.48 *	2.89 *,#	
50KG/50TA		1.28	1.74	2.12 *	3.03 *	3.96			1.13	1.47 *	2.78 *	
50KG/50TA+2%GL		1.21	1.77 ^	2.11 *,^	3.03 *,^	3.67 *,#,^			1.12	1.40 *	2.54 *,#,^	
50KG/50TA+5%GL		1.19	1.67 *,^	2.29 *,#,^	3.16 *,#,^	4.0 ^			1.16	1.50 *	2.94 *,#,^	

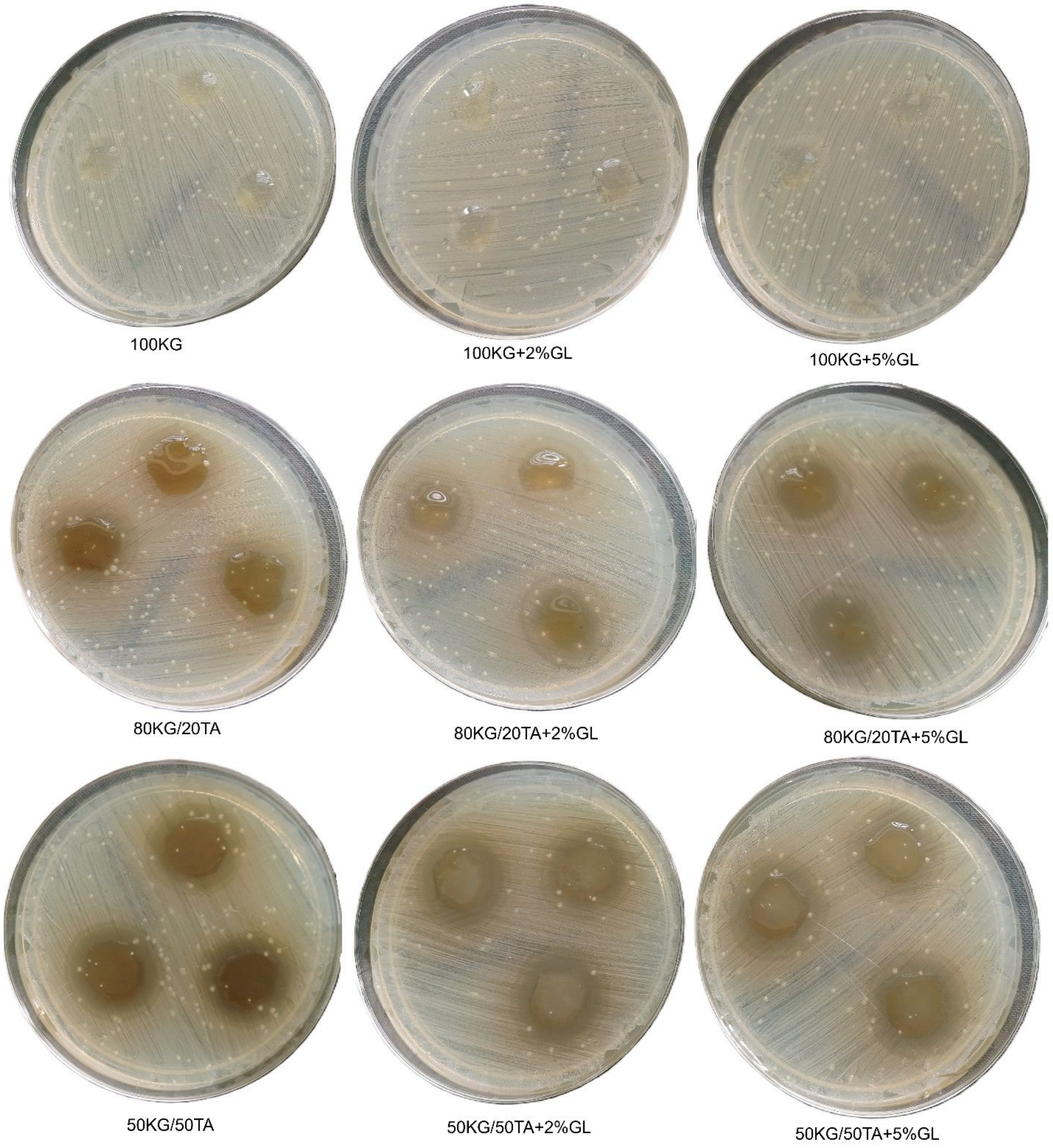

Figure S1. Antibacterial properties of films determined by the Kirby–Bauer zone of inhibition of *Staphylococcus aureus* up to seven days of incubation. The presented pictures are representative of 3 experiments.

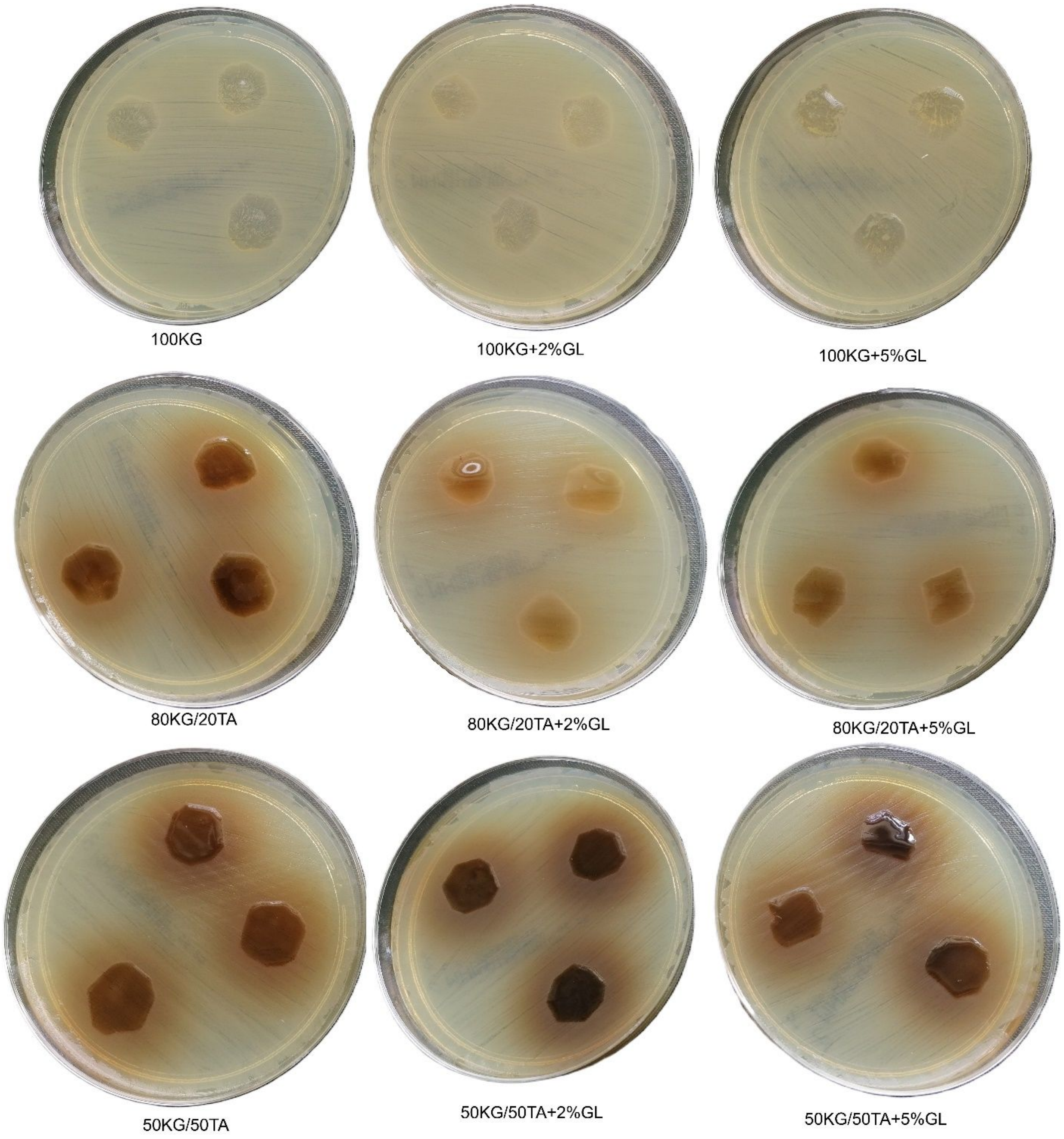

Figure S2. Antibacterial properties of films determined by the Kirby–Bauer zone of inhibition of *Escherichia coli* up to seven days of incubation. The presented pictures are representative of 3 experiments.
